# Inhibiting alpha subunit of eukaryotic initiation factor 2 dephosphorylation protects injured hepatocytes and reduces hepatocyte proliferation in acute liver injury

**DOI:** 10.3325/cmj.2019.60.532

**Published:** 2019-12

**Authors:** Guimei Chen, Xuemei Yang, Yihuai He, Yongjing Tang, Rendong Tian, Wenge Huang, Huan Chen, Fangwan Yang, Ying Li, Shide Lin

**Affiliations:** Affiliated Hospital of Zunyi Medical College, Zunyi, China; The first two authors contributed equally.

## Abstract

**Aim:**

To investigate the impact of alpha subunit of eukaryotic initiation factor 2 (eIF2α) phosphorylation on liver regeneration.

**Methods:**

Male BALB/c mice were intraperitoneally injected with carbon tetrachloride (CCl_4_) to induce liver injury. Human hepatocyte LO2 cells were incubated with thapsigargin to induce endoplasmic reticulum (ER) stress. Salubrinal, integrated stress response inhibitor (ISRIB), and DnaJC3 overexpression were used to alter eIF2α phosphorylation levels.

**Results:**

CCl_4_ administration induced significant ER stress and eIF2α phosphorylation, and increased hepatocyte proliferation proportionally to the extent of injury. Inhibiting eIF2α dephosphorylation with salubrinal pretreatment significantly mitigated liver injury and hepatocyte proliferation. In LO2 cells, thapsigargin induced significant eIF2α phosphorylation and inhibited proliferation. Inhibiting eIF2α dephosphorylation partly restored cell proliferation during ER stress.

**Conclusions:**

In acute liver injury, inhibiting eIF2α dephosphorylation protects injured hepatocytes and reduces hepatocyte proliferation.

Acute liver failure is induced by massive hepatocyte death, resulting in the loss of liver function and fatal outcome (1). Liver regeneration in response to liver injury or hepatectomy (2) can be delayed or impaired under certain circumstances. Impaired liver regeneration may delay tissue recovery, leading to poor prognosis in patients with severe liver injury. The molecular mechanisms responsible for impaired liver regeneration remain poorly understood.

The pathogenesis of a variety of liver diseases involves endoplasmic reticulum (ER) stress (3,4). ER stress is triggered by the accumulation of unfolded proteins in the ER and their binding to the ER chaperone protein glucose-regulated protein 78 (GRP78), leading to the phosphorylation of protein kinase R-like ER kinase (PERK) and activation of transcription factor 6 (ATF6) and inositol requiring enzyme 1 (IRE1) (5,6). Activated PERK phosphorylates serine 51 of the alpha subunit of eukaryotic initiation factor 2 (eIF2α). The phosphorylation of eIF2α represses protein synthesis and mitigates ER stress through reducing folding load (7). Once ER stress is attenuated, phosphorylated eIF2α may selectively induce the expression of activating transcription factor 4 (ATF4) (8), which induces the expression of growth arrest and DNA damage 34 (GADD34), GRP78, and C/EBP homologous protein (CHOP). Notably, GADD34 will interact with protein phosphatase 1 (PP1) to dephosphorylate eIF2α, which will remove protein synthesis restriction. Thus, eIF2α phosphorylation is regulated through a negative feedback loop (9).

ER stress can also be chemically regulated. For instance, salubrinal indirectly blocks eIF2α dephosphorylation by inhibiting PP1 activity, while integrated stress response inhibitor (ISRIB) inhibits eIF2α phosphorylation (10-12). In addition, DnaJC3, an ER stress-regulated chaperone, can inhibit eIF2α kinases, including PERK, protein kinase R, heme-regulated inhibitor, and general control nonderepressible 2 kinase (13,14). PERK, ATF6, and IRE1 inhibit protein synthesis, up-regulate the expression of ER response proteins, activate ER-related degradation, and promote cell survival. ER stress that disrupts ER homeostasis will activate pro-apoptotic and inflammatory signaling (15).

The phosphorylation of eIF2α is known to mitigate liver injury (16). However, its regulatory impact on liver regeneration in acute liver injury has yet to be established. In this study, we investigated the effect of eIF2α phosphorylation on hepatocyte proliferation to propose a strategy for acute liver injury prevention.

## Materials and methods

### Animals and induction of liver injury

Male BALB/c mice (6-8 weeks old, 18 ± 2 g), supplied by the Animal Center of Zunyi Medical College (Guizhou, China), were housed in a specific pathogen-free facility at a temperature between 20-24°C and maintained on a 12-h light/dark cycle in the Animal Center of Zunyi Medical College (Guizhou, China). Mice were acclimated for one week before experimental procedures. All animal studies were carried out in accordance with the guidelines of China Animal Care and Research. The animal study protocol was approved by the Animal Care and Use Committee of the Affiliated Hospital to Zunyi Medical University (ZMC · LS [2018]28).

A total of 240 mice were randomly divided into 15 groups using a random number table (Table 1) (17). To induce acute liver injury, mice were injected intraperitoneally with 10 mL/kg body weight of a mixture of CCl_4_ (25%, carbon tetrachloride) and olive oil (75%) at the doses of 2, 10, or 20 mL/kg. Control mice were injected with 10 mL/kg body weight of olive oil alone. To investigate the regulatory impact of eIF2α phosphorylation on hepatocyte proliferation during acute liver injury, eIF2α phosphorylation levels in mice were altered with salubrinal, ISRIB, and DnaJC3 overexpression pretreatment. The salubrinal + CCl_4_ group was pretreated with an intraperitoneal injection of salubrinal (1 mg/kg body weight; vehicle: dimethyl sulfoxide [DMSO]; Sigma Aldrich, St. Louis, MO, USA) and then injected with CCl_4_. ISRIB + CCl_4_ group was pretreated with an intraperitoneal injection of ISRIB (2.5 mg/kg body weight; vehicle: phosphate buffer solution [PBS]; Sigma Aldrich) for 2 h, then injected with CCl_4_. Salubrinal group was injected with the same dose of salubrinal, followed by olive oil, while ISRIB group was injected with ISRIB and olive oil. DnaJC3 + CCl_4_ control group was pretreated via the tail vein with a recombinant adeno-associated virus serotype 8 that expressed DnaJC3 (rAAV8-DnaJC3, NM-008929, Genechem, Shanghai, China) and injected with CCl_4_ four weeks later. AAV8 + CCl_4_ control group was pretreated with AAV8 (2 × 10^10^ v.g. in 200 μL PBS per mouse) and injected with CCl_4_ four weeks later.

**Table 1:** Animal groups and treatment*

Group	Pretreatment	Treatment	Time of sacrifice	Mice per group	Total mice
Time-dependent induced acute liver injury (98 mice)					
CCl_4_	no	CCl_4_	0.5, 1, 2, 3, 4, and 5 days after injection	8	56
Control	no	olive oil	0.5, 1, 2, 3, 4, and 5 days after injection	6	42
Dose-dependent induced acute liver injury (32 mice)					
Control	no	olive oil	1 day	8	8
2 mL/kg CCl_4_	no	CCl_4_	1 day	8	8
10 mL/kg CCl_4_	no	CCl_4_	1 day	8	8
20 mL/kg CCl_4_	no	CCl_4_	1 day	8	8
Salubrinal, ISRIB, or overexpressed DnaJC3-altered eIF2α phosphorylation (110 mice)					
Untreated	no	no	1 day	6	6
Salubrinal	salubrinal	olive oil	1 day	6	6
ISRIB	ISRIB	olive oil	1 day	6	6
Control AAV8	control AAV8	olive oil	1 day	6	6
rAAV8-DnaJC3	DnaJC3	olive oil	1 day	6	6
CCl_4_	PBS	CCl_4_	1 and 3 day	8	16
Salubrinal + CCl_4_	salubrinal	CCl_4_	1 and 3 day	8	16
ISRIB + CCl_4_	ISRIB	CCl_4_	1 and 3 day	8	16
AAV8 + CCl_4_	control AAV8	CCl_4_	1 and 3 day	8	16
DnaJC3 + CCl_4_	DnaJC3	CCl_4_	1 and 3 day	8	16

*AAV8 – adeno-associated virus serotype 8; CCl_4_ – carbon tetrachloride ; PBS – phosphate buffer saline; ISRIB – integrated stress response inhibitor; rAAV8: – recombinant AAV8.

### Cell culture and endoplasmic reticulum stress induction

The human hepatocyte cell line LO2 was obtained from the Cell Bank of the Type Culture Collection at the Chinese Academy of Sciences (Shanghai, China). LO2 cells were cultured in RPMI 1640 with 10% fetal bovine serum and 1% penicillin/streptomycin. To investigate the effects of eIF2α phosphorylation on cell survival under ER stress, LO2 cells were pretreated with salubrinal (20 μM, Sigma) or ISRIB (2.5 μM, Sigma) for 2 h, then treated with DMSO (control) or thapsigargin (TG, l μM, Sigma).

### Western blot analysis

To investigate the role of eIF2α phosphorylation on acute liver injury, the relative protein levels of phosphorylated eIF2α (p-eIF2α), eIF2α, and CHOP were determined by Western blot. Autopsied liver tissues were homogenized (10 mg/mL) upon sacrifice. After centrifuging the homogenates, individual liver lysates or cell lysates (40 μg/lane) were separated using 10%-12% sodium dodecyl sulfate polyacrylamide gel electrophoresis and transferred to polyvinylidene fluoride membranes (Millipore, Billerica, MA, USA). The membranes were blocked with 5% fat-free dry milk in Tris-buffered saline with Tween 20 (TBST) and probed with mouse monoclonal antibodies against β-actin (sc-58673, sc: Santa Cruz Biotechnology, Dallas, TX, USA), cyclin D1 (sc-56302), CHOP (ab11419, ab: Abcam), eIF2α (sc-133132), and proliferating cell nuclear antigen (PCNA, sc-25280), and rabbit monoclonal antibodies against DnaJC3 (MA5-14820, Thermo Fisher Scientific, Waltham, MA, USA) and phosphorylated eIF2α (p-eIF2α, 3398, Cell Signaling Technology, Danvers, MA, USA). After washes in TBST, the bound antibodies were detected with horseradish peroxidase-conjugated anti-mouse or anti-rabbit IgG and visualized using enhanced chemiluminescent reagents. The relative level of each target protein to the control was densitometrically determined using Quantity One software (Bio-Rad, Hercules, CA, USA).

### Cell viability assay

Cell viability was assessed using the MTS [3-(4,5-dimethylthiazol-2-yl)-5-(3-carboxymethoxyphenyl)-2-(4-sulfophenyl)-2H-tetrazolium] Cell Titer 96^®^ AQueous One Solution Cell Proliferation assay kit (Promega Corporation, Madison, WI, USA) according to the manufacturer’s instructions. Cell viability was determined by replacing the medium with 20 μL of MTS. After incubating the cells at 37°C for 3 h, absorbance was measured at 490 nm using a microplate reader (Bio-Rad model 680; Bio-Rad). Cell viability was normalized as a percentage of control. This experiment was repeated five times.

### Histology and immunohistochemistry

Liver tissues were fixed in 10% formalin and embedded in paraffin. Sections (5 μm thickness) were stained with hematoxylin and eosin. In addition, the tissue sections were subjected to immunohistochemistry using monoclonal antibodies against PCNA (sc-25280) or p-eIF2α (3398, Cell Signaling Technology). The sections were imaged under a light microscope.

### Serum alanine aminotransferase and total bilirubin level

Terminal blood samples were collected from each animal upon euthanasia. Serum alanine aminotransferase (ALT) and total bilirubin levels were determined with use of the Beckman Coulter auto-analyzer (AU5800, Beckman Coulter, Brea, CA, USA).

### Statistical analysis

The normality of distribution was tested with the one-sample Kolmogorov-Smirnov test. Data are presented as median with range and the groups were compared with the Kruskal-Wallis test with Mann-Whitney U test *post hoc* analysis. The level of significance was set at *P* < 0.05. Statistical analysis was conducted in SPSS 18.0 (SPSS Inc., Chicago, IL, USA).

## Results

### CCl_4_ injection induces liver injury, intrahepatic ER stress, and hepatocyte proliferation in mice

**Figure 1:**
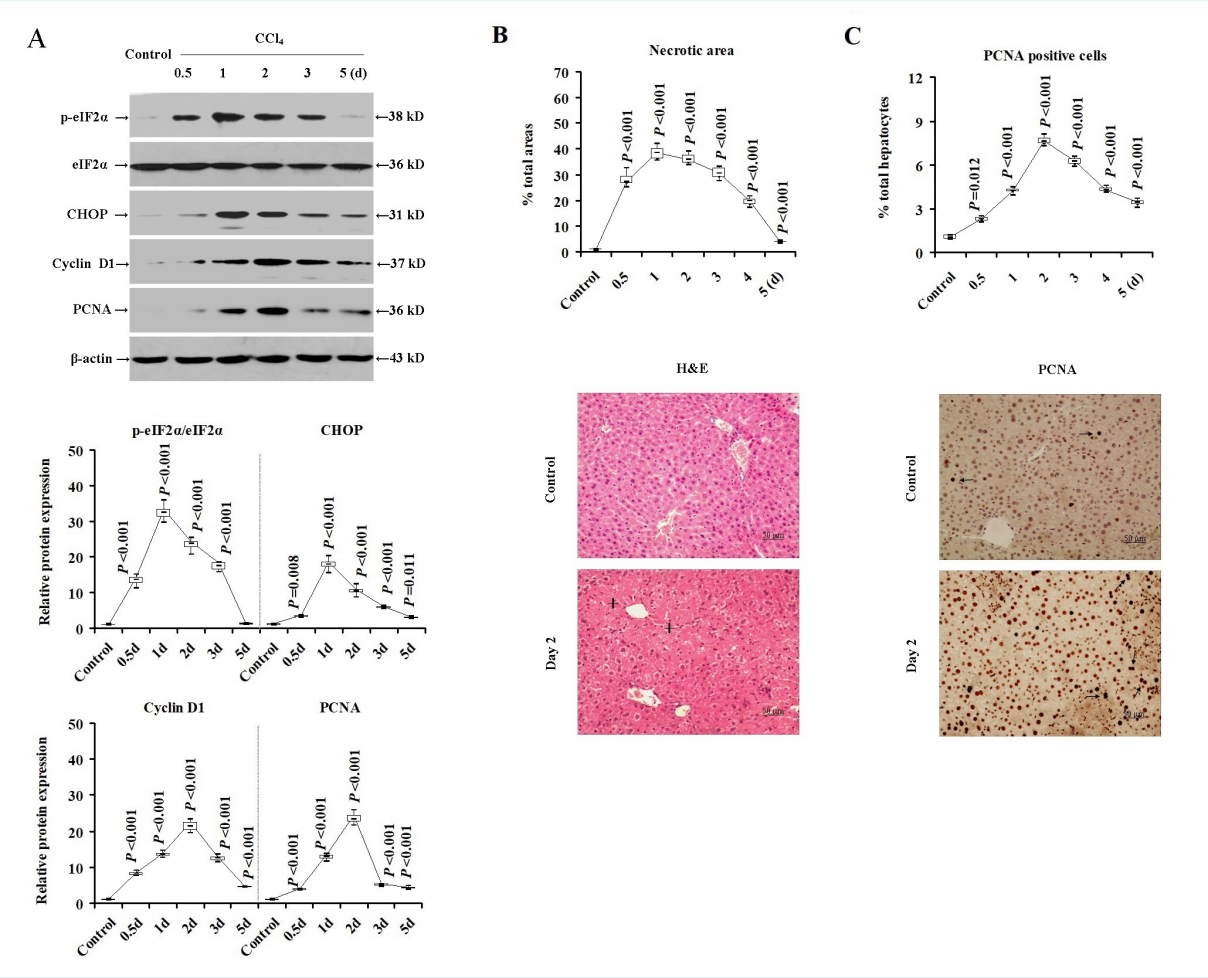
Administration of carbon tetrachloride (CCl_4_) induces liver injury, intrahepatic endoplasmic reticulum stress, and hepatocyte proliferation in mice. Male BALB/c mice were randomly distributed to a group injected with a mixture of olive oil and CCl_4_ (n = 8, total mice = 56) or a group injected with olive oil (control; n = 6, total mice = 42). (**A**) The time-dependent effect on intrahepatic alpha subunit of eukaryotic initiation factor 2 (eIF2α), phosphorylated eIF2α (p-eIF2α), cyclin D1, and proliferating cell nuclear antigen (PCNA) expression determined by Western blot at 0.5, 1, 2, 3, 4, and 5 days after CCl_4_ or olive oil injection. (**B**) Hepatocyte necrosis in hematoxylin and eosin (H&E)-stained sections. Plus indicates the necrotic area. (**C**) Immunohistochemistry analysis of intrahepatic PCNA expression (magnification ×100). *P*-value, compared with the control group. Arrow indicates PCNA-positive cells.

In our preliminary study, intrahepatic ER stress and hepatocyte proliferation in mice mainly occurred 1-2 days after CCl_4_ injection. CCl_4_ administration significantly increased serum ALT (Table 2) and total bilirubin (Table 2), and induced intrahepatic eIF2α phosphorylation, CHOP, cyclin D1, PCNA protein expression (Figure 1A), and hepatocyte necrosis (Figure 1B). Phosphorylation of eIF2α and CHOP protein expression peaked at day 1, and cyclin D1 and PCNA expression peaked at day 2 after CCl_4_ injection. A similar PCNA expression pattern was detected by immunohistochemistry, showing hepatocyte proliferation occurred in the remaining normal liver tissue (Figure 1C). These data clearly demonstrate that CCl_4_ injection induces liver injury and intrahepatic ER stress, as well as eIF2α phosphorylation, triggering hepatocyte proliferation in mice.

**Table 2:** The effects of carbon tetrachloride (CCl_4_) on serum alanine aminotransferase (ALT) activity and bilirubin concentration

	Control	CCl_4_	P*
ALT (median [25%-75%], U/L)			
Day 0	36.5 (33.75-37.75)	41 (38.75-44.75)	0.59
Day 0.5	42 (40.25-43.75)	2616.5 (2261.25-3001.75)	<0.001
Day 1	50.5 (46.5-53)	4669.5 (4161.25-4937.25)	<0.001
Day 2	51.5 (48-52.75)	5156 (4683.75-5483)	<0.001
Day 3	51 (47-60.25)	1526.5 (1289.5-1631.25)	<0.001
Day 4	54 (47.5-57.5)	270.5 (246.75-277.25)	0.002
Day 5	51.5 (46.25-55.25)	88 (83.75-91.5)	0.058
Total bilirubin (median [25%-75%], μmol/L)			
Day 0	1.05 (0.925-1.175)	1.05 (0.9-1.2)	0.95
Day 0.5	1.3 (1.2-1.4)	1.55 (1.475-1.65)	0.2
Day 1	1.2 (1.125-1.275)	3.05 (2.9-3.225)	0.002
Day 2	0.95 (0.825-1)	5.15 (4.875-5.225)	<0.001
Day 3	1.2 (1.125-1.2)	5.75 (5.475-6.2)	<0.001
Day 4	1.05 (0.925-1.175)	1.05 (0.9-1.2)	<0.001
Day 5	1.3 (1.2-1.4)	1.55 (1.475-1.65)	0.03

*CCl_4_ compared with the control group.

### Severe liver injury stimulates hepatocyte proliferation

**Figure 2:**
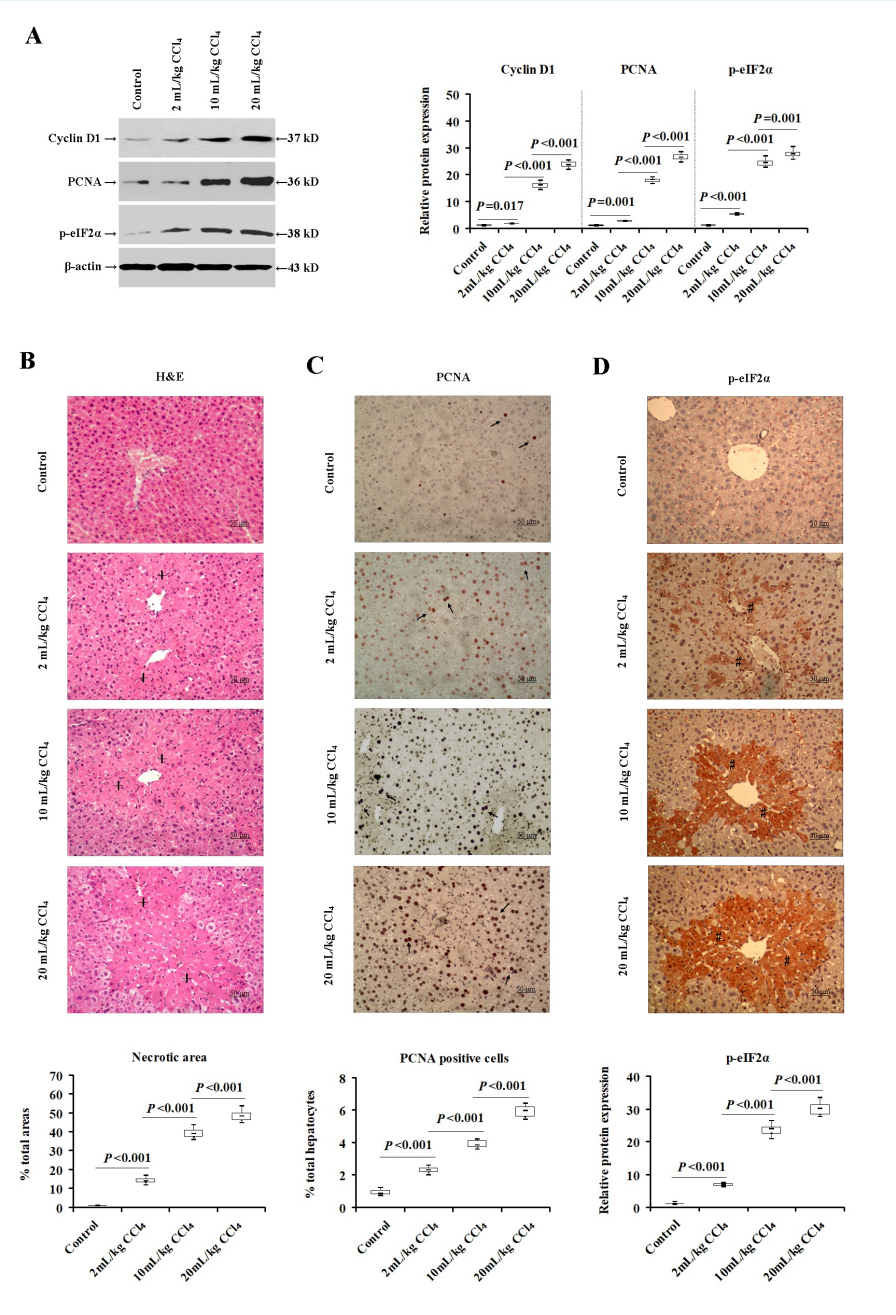
Severe liver injury stimulates hepatocyte proliferation. Male BALB/c mice were injected with 10 mL/kg body weight of olive oil alone (control) or a mixture of carbon tetrachloride (CCl_4_) and olive oil at doses of 2 (low dose), 10 (medium dose), or 20 (high dose) mL/kg (n = 8, total mice = 32). (**A**) The dose-dependent effect on intrahepatic cyclin D1, proliferating cell nuclear antigen (PCNA), and phosphorylated alpha subunit of eukaryotic initiation factor 2 (p-eIF2α) expression determined by Western blot. (**B**) Hepatocyte necrosis in hematoxylin and eosin (H&E)-stained sections. Plus indicates the necrotic area. (**C**) Immunohistochemistry analysis of intrahepatic PCNA and (**D**) phosphorylated alpha subunit of eukaryotic initiation factor 2 (p-eIF2α) expression (magnification ×100). Arrow indicates PCNA positive cells. Hash sign indicates the positive area.

Both serum ALT and total bilirubin levels were elevated in a CCl_4_ dose-dependent manner (Table 3), while intrahepatic cyclin D1, PCNA, p-eIF2α protein expression (Figure 2A), and hepatocyte necrosis (Figure 2B) were increased 1 day after injection. Similar PCNA (Figure 2C) and p-eIF2α expression patterns were detected by immunohistochemistry in the injured liver tissues (Figure 2D). These data demonstrate that ER stress was triggered by liver injury, as evidenced by eIF2α phosphorylation. Furthermore, they indicate that hepatocyte proliferation is proportional to the extent of injury in mice.

**Table 3:** Dose-dependent effect of carbon tetrachloride (CCl_4_) on serum alanine aminotransferase (ALT) and total bilirubin

	ALT (median [25%-75%], U/L)	Total bilirubin (median [25%-75%], μmol/L)
Control	37.5 (30.5-39.25)	1 (0.875-1.1)
2 mL/kg CCl_4_	2850 (2543.5-3087.5)	1.5 (1.375-1.625)
10 mL/kg CCl_4_	4537 (4295.5-4771.25)	3.45 (3.375-3.625)
20 mL/kg CCl_4_	7122 (6702.25-7278)	4.95 (4.775-5.125)
χ^2^	29.091	29.027
*P*	<0.001	<0.001

### Inhibiting eIF2α dephosphorylation mitigates CCl_4_-induced hepatocyte proliferation

**Figure 3:**
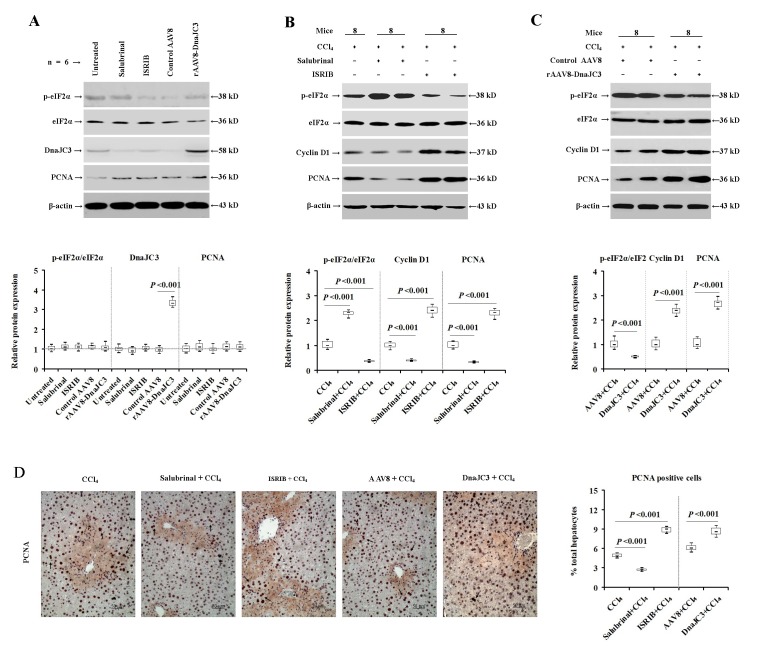
The inhibition of alpha subunit of eukaryotic initiation factor 2 (eIF2α) dephosphorylation mitigates carbon tetrachloride (CCl_4_)-induced hepatocyte proliferation in mice. Male BALB/c mice were pretreated with vehicle (dimethyl sulfoxide + phosphate buffer solution), salubrinal, integrated stress response inhibitor (ISRIB) for 2 h, or recombinant AAV8 expressing DnaJC3 for 4 weeks, then injected with olive oil or CCl_4_. Relative levels of intrahepatic cyclin D1 and proliferating cell nuclear antigen (PCNA) expression at 24 h after CCl_4_ injection were determined by Western blot. PCNA expression was assessed by immunohistochemistry. (**A**) Salubrinal, ISRIB, or overexpressed DnaJC3 altered eIF2α phosphorylation, DnaJC3, and PCNA expression (n = 6; total mice = 30). (**B**) Salubrinal, ISRIB, or (**C**) overexpressed DnaJC3 altered the levels of CCl_4_-induced eIF2α phosphorylation, cyclin D1, and PCNA expression (n = 8, total mice = 40, day 1). (**D**) Immunohistochemistry analysis of hepatic PCNA expression (magnification ×100).

Salubrinal, ISRIB, or DnaJC3 overexpression treatment did not alter serum ALT levels (Table 4), intrahepatic eIF2α phosphorylation, and PCNA protein expression (Figure 3A) in mice without liver injury. Salubrinal significantly increased CCl_4_-induced eIF2α phosphorylation, and reduced cyclin D1 and PCNA protein expression. ISRIB and DnaJC3 overexpression significantly reduced eIF2α phosphorylation, and increased cyclin D1 and PCNA protein expression (Figure 3B and 3C). A similar pattern of intrahepatic PCNA expression was also detected using immunohistochemistry (Figure 3D). Collectively, these data indicate that inhibiting eIF2α dephosphorylation mitigates hepatocyte proliferation during acute liver injury.

**Table 4:** The effect of salubrinal, ISRIB, or overexpressed DnaJC3 on serum alanine aminotransferase (ALT) activity*

	ALT (median [25%-75%], U/L)
Untreated	44 (39.75-46.75)
Salubrinal	42.5 (40.5-44.5)
ISRIB	42.5 (39.75-43.75)
Control AAV8	42 (40.25-44.5)
rAAV8-DnaJC3	43.5 (40-44.75)
χ^2^	0.263
*P*	0.996

*AAV8 – adeno-associated virus serotype 8; ISRIB – integrated stress response inhibitor; rAAV8: – recombinant AAV8.

### Inhibiting eIF2α dephosphorylation moderates ER stress-related apoptosis and hepatocyte injury in response to CCl_4_ injury

**Figure 4:**
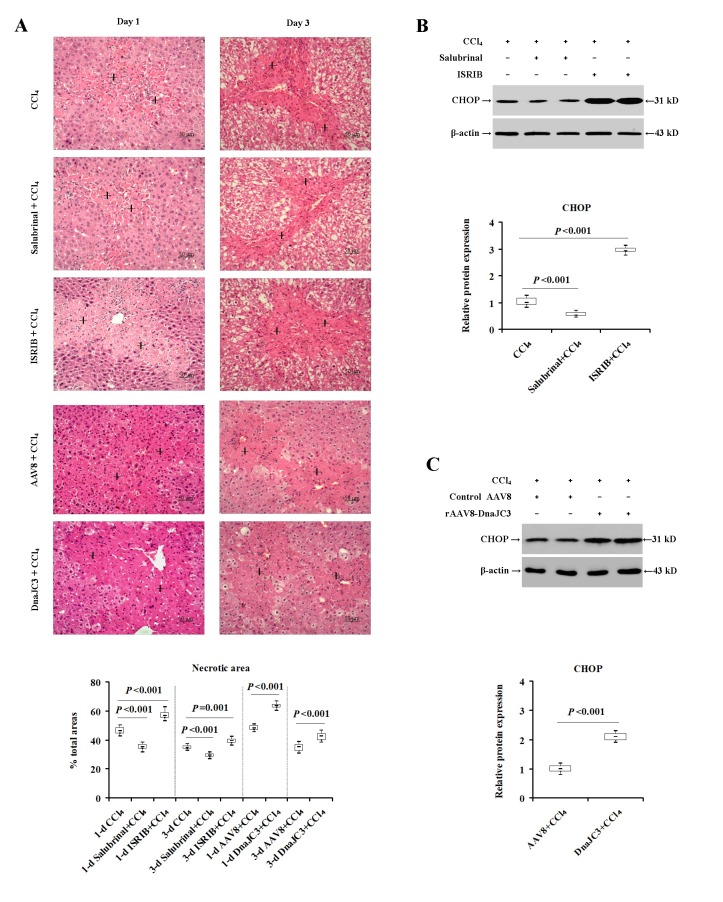
The inhibition of alpha subunit of eukaryotic initiation factor 2 (eIF2α) dephosphorylation mitigates endoplasmic reticulum-related apoptosis and hepatocyte injury in response to carbon tetrachloride (CCl_4_) injury. Male BALB/c mice were pretreated with vehicle, salubrinal, or integrated stress response inhibitor (ISRIB) for 2 h, or with the recombinant AAV8 that expressed DnaJC3 for 4 weeks, then injected with olive oil or CCl_4_ (n = 8, total mice = 40; day 3). (**A**) Liver histology. Plus indicates the necrotic area. (**B**) Salubrinal, ISRIB, or (**C**) overexpressed DnaJC3 altered the levels of C/EBP homologous protein (CHOP).

In contrast to the pretreatment with ISRIB and DnaJC3, salubrinal pretreatment significantly reduced CCl_4_-induced serum ALT (Table 5), total bilirubin (Table 5), hepatocyte necrosis (Figure 4A), and intrahepatic CHOP protein expression (Figure 4B and 4C). Taken together, these data indicate that inhibiting eIF2α dephosphorylation moderates CCl_4_-induced ER stress-related apoptosis and liver injury_._

**Table 5:** The effect of salubrinal, ISRIB, or overexpressed DnaJC3 on serum alanine aminotransferase (ALT) activity and total bilirubin

	Day 1 (median [25%-75%], U/L)	Day 3 (median [25%-75%], U/L)
ALT		
CCl_4_	4792.5 (4341.75-4959.5)	1498 (1301-1708.5)
Salubrinal + CCl_4_	3468.5 (3201.75-3871.5)^†^	1022.5 (850.5-1088.75)^†^
ISRIB + CCl_4_	5676 (5544-5898.75)^†^	2208.5 (1967.75-2353.25)^†^
AAV8 + CCl_4_	5708.5 (5484.75-5877.5)	1424.5 (1328.75-1560.5)
DnaJC3 + CCl_4_	6735.5 (6478-6884.5)^‡^	2007.5 (1874.5-2181.5)^‡^
Bilirubin		
CCl_4_	3.35 (3.075-3.525)	5.25 (5.075-5.525)
Salubrinal + CCl_4_	2.5 (2.375-2.625)^†^	4.3 (4.05-4.525)^†^
ISRIB + CCl_4_	3.95 (3.775-4.125)^†^	6.65 (6.35-6.825)^†^
AAV8 + CCl_4_	3.1 (2.975-3.225)	4.75 (4.375-4.95)
DnaJC3 + CCl_4_	4.55 (4.35-4.725)^‡^	6 (5.75-6.325)^‡^

*AAV8 – adeno-associated virus serotype 8; CCl_4_ – carbon tetrachloride; PBS – phosphate buffer saline; ISRIB – integrated stress response inhibitor; rAAV8: – recombinant AAV8.

†*P* < 0.01 vs the CCl_4_ group.

‡*P* < 0.01 vs the AAV8 + CCl_4_ group.

### Inhibiting eIF2α dephosphorylation partly restores TG-inhibited proliferation in LO2 cells

**Figure 5:**
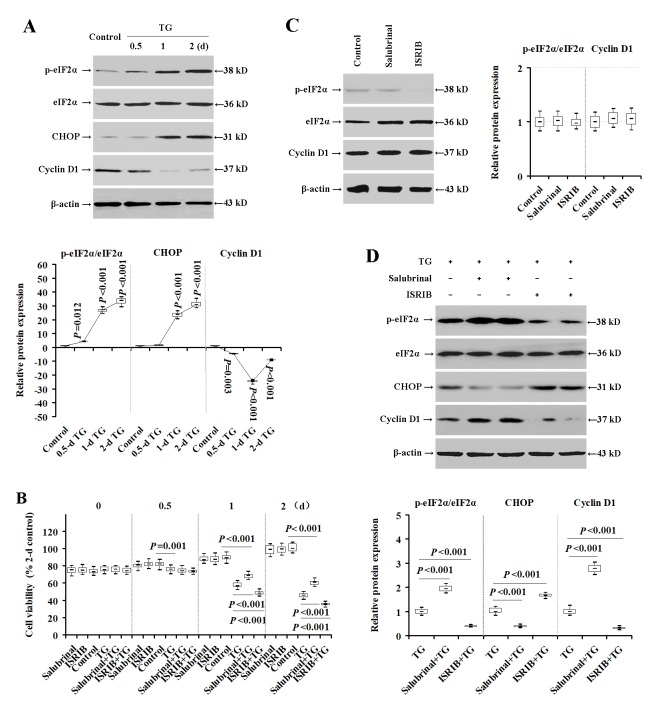
The inhibition of alpha subunit of eukaryotic initiation factor 2 (eIF2α) dephosphorylation partly restores thapsigargin (TG)-inhibited proliferation in LO2 cells. LO2 cells were pretreated with vehicle (dimethyl sulfoxide [DMSO] + phosphate buffer solution), salubrinal, or integrated stress response inhibitor (ISRIB) for 2 h, and further treated with DMSO or TG. (**A**) eIF2α phosphorylation, C/EBP homologous protein (CHOP), and cyclin D1 protein expression at different time points after TG treatment in LO2 cells. *P*-value, compared with the control group. (**B**) Viability of LO2 cells determined by MTS. Data from three independent experiments are presented. (**C**) Relative protein expression of phosphorylated eIF2α (p-eIF2α), eIF2α, and cyclin D1 in the control-, salubrinal-, or ISRIB-treated LO2 cells. (**D**) eIF2α phosphorylation, CHOP, and cyclin D1 protein expression at 24 h after TG treatment in different groups.

TG treatment of LO2 cells significantly increased eIF2α phosphorylation and CHOP protein expression, and reduced cyclin D1 protein expression (Figure 5A) and cell viability (Figure 5B). Neither salubrinal nor ISRIB incubation significantly affected cell viability, eIF2α phosphorylation, or cyclin D1 protein expression (Figure 5C) in LO2 cells without ER stress. However, in contrast with ISRIB pretreatment, salubrinal pretreatment significantly increased TG-induced eIF2α phosphorylation, reduced CHOP protein expression, and partly restored cyclin D1 protein expression (Figure 5D) and cell viability. The partly restored cell viability was significantly lower compared with control LO2 cells. These data indicate that ER stress inhibits hepatocyte proliferation and that inhibiting eIF2α dephosphorylation promotes hepatocyte survival and partly restores cell proliferation during ER stress.

## Discussion

In this study, CCl_4_ administration induced acute liver injury and hepatic eIF2α phosphorylation, followed by liver cell proliferation. Inhibiting eIF2α dephosphorylation mitigated the CCl_4_-induced liver injury, as well as the extent of liver cell division in the non-injured parenchyma. These results suggest that if eIF2α dephosphorylation is inhibited during acute liver injury, the survival of the remaining normal hepatocytes may increase and the hepatocyte proliferative response as a result of reduced liver injury may decrease. Moreover, in LO2 cells, TG-induced ER stress-related apoptosis was moderated and cell proliferation partly restored by inhibiting eIF2α dephosphorylation, showing that the inhibition of eIF2α dephosphorylation may as well confer resistance to CCl_4_ insult, enabling the liver cells to survive.

Hepatocyte proliferation is required for liver regeneration after liver injury, and active hepatocyte division can effectively repair the damaged liver (18,19) and restore hepatic function and structure (20). CCl_4_ induces liver injury by facilitating hepatic lipid peroxidation and oxidative stress, which lead to ER stress and liver cell injury as well as eIF2α phosphorylation (21). In this study, higher CCl_4_ doses induced more severe liver injury and greater cell proliferation. Moreover, CCl_4_-induced hepatocyte proliferation occurred in the remaining normal liver tissue. This is consistent with the results of other studies, which showed that hepatocytes, both mature and quiescent, proliferate in response to liver injury (22). These results suggest that severe liver injury stimulated normal hepatocyte proliferation *in vivo*.

ER stress is a compensatory protective mechanism in response to injury, which, if excessive or sustained, can activate apoptosis and inflammation (23). ER stress-related apoptosis is mediated by the CHOP, caspase-12, and c-Jun N-terminal kinase pathways (24). ER stress (16,25) and stress-induced cellular injury (26) can be mitigated by eIF2α phosphorylation, which reduces the rate of protein synthesis. Salubrinal selectively blocks eIF2α dephosphorylation by inhibiting PP1 activity (27). In this study, eIF2α was dephosphorylated in control mice and cells. Salubrinal, ISRIB, and DnaJC3 overexpression did not cause eIF2α phosphorylation alone, but in combination with ER stress. In addition, eIF2α phosphorylation mainly occurred in injured areas, suggesting that it is associated with liver injury. Salubrinal, ISRIB, and DnaJC3 do not induce liver injury and ER stress; therefore, they cannot elevate eIF2α phosphorylation in the liver without injury. Inhibiting eIF2α dephosphorylation moderated CCl_4_-induced hepatocyte proliferation, mainly around the intrahepatic injured area, possibly through relieving hepatocyte loss and reducing the need for liver regeneration due to reduced liver injury.

The phosphorylation of eIF2α represses protein synthesis and initiates ER stress gene expression, which involve both pro-survival and pro-apoptotic pathways of ER stress (28). We found that CCl_4_ administration induced ER stress and CHOP protein expression *in vivo*. CCl_4_-induced CHOP protein expression was reduced and liver injury in mice was moderated by selective inhibition of eIF2a dephosphorylation. These results suggest that inhibiting eIF2α dephosphorylation reduces ER stress-related apoptosis and protects hepatocytes from injury, thereby reducing the need for hepatocyte proliferation after liver injury.

Hepatocyte proliferation, required for liver regeneration after liver injury (29), can be arrested by ER stress through a variety of mechanisms (30). TG disturbs ER calcium homeostasis and induces ER stress and eIF2α phosphorylation (31). In this study, TG induced eIF2α phosphorylation and ER stress-related apoptosis, as well as inhibited LO2 cell proliferation. ER stress-related apoptosis was reduced and proliferation and cell viability in LO2 cells was partly restored by inhibiting eIF2α dephosphorylation. However, the partly restored cell proliferation was significantly reduced compared with control LO2 cells.

Net liver regeneration after liver injury is largely determined by the relative ratio of hepatocyte proliferation and death (32,33). Inhibiting eIF2α dephosphorylation significantly reduced hepatocyte necrosis and apoptosis, and increased the survival of the remaining normal hepatocytes, although hepatocyte proliferation was still reduced. Taken together, inhibiting eIF2α dephosphorylation prolonged hepatocyte survival, prevented hepatocyte loss, and reduced hepatocyte proliferation during acute liver injury.

One of the limitations of the present study was that eIF2α dephosphorylation was chemically regulated. The compounds used may also act on other cells or affect study findings in other ways. The potentially confounding effects can be reduced if eIF2α dephosphorylation occurs at the transcriptional level in mice after acute liver injury. Secondly, only cyclin D1 and PCNA were used to reflect the regeneration of liver cells, thus not covering all regenerative events. Despite the limitations, this is the first study to reveal that the inhibition of eIF2α dephosphorylation mitigates acute liver injury, representing a viable strategy to moderate acute liver injury.
